# Sea Bass Side Streams Extracts Obtained by Pulsed Electric Fields: Nutritional Characterization and Effect on SH-SY5Y Cells

**DOI:** 10.3390/foods12142717

**Published:** 2023-07-16

**Authors:** Francisco J. Martí-Quijal, Juan Manuel Castagnini, María-José Ruiz, Francisco J. Barba

**Affiliations:** 1 Research Group in Innovative Technologies for Sustainable Food (ALISOST), Preventive Medicine and Public Health, Food Science, Toxicology and Forensic Medicine Department, Faculty of Pharmacy, Universitat de València, Avda. Vicent Andrés Estellés, s/n, 46100 Burjassot, Spain; francisco.j.marti@uv.es (F.J.M.-Q.); francisco.barba@uv.es (F.J.B.); 2 Research Group in Alternative Methods for Determining TOXICS Effects and Risk Assessment of Contaminants and Mixtures (RiskTox), Preventive Medicine and Public Health, Food Science, Toxicology and Forensic Medicine Department, Faculty of Pharmacy, Universitat de València, Avda. Vicent Andrés Estellés, s/n, 46100 Burjassot, Spain; m.jose.ruiz@uv.es

**Keywords:** pulsed electric fields, fish side streams, antioxidants, minerals, heavy metals, bioactive peptides, cell viability, SH-SY5Y

## Abstract

Fish side streams are an environmental and economic problem. In this work, pulsed electric fields (PEF) extraction was optimized and used as a new method for their valorization. Sea bass head, skin, viscera, and backbone were used. PEF technology (123–300 kJ/kg, 1–3 kV/cm) improved the extraction of proteins and antioxidant compounds from head and skin, but was not successful for viscera. SDS-PAGE showed that the protein molecular weight distribution was affected by the extraction process, revealing differences between the control and PEF extraction conditions. In addition, the extraction of macro-minerals and micro-minerals were also evaluated. The effect of PEF differed according to the matrix and the mineral studied. Heavy metals were also taken into account, studying the presence of As, Cd, Hg, and Pb in the extracts. PEF pre-treatment reduced the presence of As in skin, viscera, and backbone, ranging from 18.25 to 28.48% according to the matrix evaluated. The analysis of potential antioxidant bioactive peptides showed that the treatment of the sample directly influenced their variety. Additionally, the extracts obtained from the head were found to increase cell viability when tested on SH-SY5Y cells. In conclusion, PEF extraction can be a useful tool for the valorization of fish side streams.

## 1. Introduction

Recently, the European Commission adopted a circular economy action plan as a part of the European Green Deal. Among others, it is designed to promote circular economy processes and to ensure that waste is prevented and the resources used are kept in the EU economy for as long as possible [1].

The fishing industry would be a good starting point to tackle this strategy, considering several factors, such as: (i) the increase in consumption and the expansion of fish production over the last decades; (ii) the large amount of waste generated; and (iii) the potential commercial interest of this waste. The latest estimates indicate that global fish production reached about 178 million tonnes in 2020 and consumption increased at an average annual rate of 3.0% between 1961 and 2019 [2]. The increase in fish consumption and production in recent decades can be attributed to various factors, including economic development, technological advancements, expansion of aquaculture, and supportive government policies, among others [2]. The fishing industry can align with the circular economy action plan by adopting sustainable practices, minimizing waste, and utilizing side streams. Embracing resource efficiency and innovative technologies further contributes to a more sustainable and resource-efficient economy.

Fish processing side streams can range between 30% to 70% of the fresh weight (differing according to the fish species). In this sense, muscle cuts represent 15–20%, while other products are also discarded as a low-value material, such as viscera, bones, head, skin, and fins, representing 12–18%, 9–15%, 9–12%, and 1–3%, respectively [3]. These sides streams have been traditionally used primarily to produce fishmeal and fish oil for animal feeding. However, this is a small portion (currently estimated at 27–48%) and most of the side streams are discarded [2].

With these data in mind, it is necessary to drive our attention to the development of additional valorization approaches and the research about their beneficial properties that could enhance the minimization of this fish side stream while, at the same time, higher economic benefits are achieved and environmental impact reduction is promoted. However, it should be considered that the choice of the extraction method for these side streams is just as important as the valorization of the side streams themselves. In the context of the European Green Deal, much effort has been devoted to developing more sustainable and environmentally friendly extraction processes. These innovative approaches, so-called “green technologies”, reduce some of the limitations associated with conventional extraction methods by requiring less time, energy, and solvent, thus mitigating the impact on the environment [4,5,6].

Several studies have been focused on the utilization of fish side streams through the application of green technologies [6,7,8]. Specifically, pulsed electric fields (PEF) is a technology based on the application of short electrical pulses at high voltage for brief periods and have been demonstrated to be a sustainable alternative to conventional extraction methods [4,5]. Some applications of PEF in fish side streams can be found in the literature, confirming their potential to be utilized as an ecologically acceptable and cost-effective tool to valorize them [6,8]. Specifically, in our research group, PEF has been applied to trout and sole side streams to obtain extracts with antioxidant [9], antibacterial, and anti-inflammatory activities [10].

In the framework of the European community, sea bass (*Dicentrarchus labrax*) is one of the most widely consumed products, as well as one of the six most farmed species [11]. Accordingly, both the characterization and utilization of sea bass side streams have attracted recent research interest. According to the chemical composition analysis of the bass skin, guts, gills, liver, head, and bones of sea bass, they are an interesting source of proteins, minerals (calcium and phosphorus), and lipids. The lipid fraction contains high amounts of unsaturated fatty acids (particularly monounsaturated and omega-3 fatty acids) [12]. In addition, our research group has obtained antioxidant compounds through the fermentation of sea bass side streams by bacteria isolated from their fish viscera [13]. All of these nutrients and bioactive compounds have the potential to be utilized in the development of food supplements or enriched foods, which could contribute to enhancing the health and well-being of people.

On the other hand, other techniques have been used for the valorization of sea bass ide streams, obtaining extracts with antioxidant capacity through the application of ultrasound-assisted extraction (UAE) [14] and pressurized liquid extraction (PLE) [15]. Specifically, studies on nutritional value have been carried out. For instance, it has been shown that protein recovery percentages of 7–28% (muscle), 11–40% (heads), 26–99% (viscera), 7–34% (skin), 24–75% (bones), and 10–30% (tails) were obtained after the application of green technologies, such as UAE [14] and PLE [15].

Regarding pulsed electric fields (PEF), several researchers have used this technology to recover various compounds from fish side streams. For instance, Burnett et al. [16] used PEF to extract lipids from Hoki male gonad. In their study, an electric field strength ranging from 0.625 to 1.875 and a frequency between 25 and 100 Hz was applied. They found that the highest lipid extraction yield was obtained at 1.25 kV/cm and 50 Hz. Similarly, Wang et al. [9] used PEF to extract proteins and antioxidant compounds from rainbow trout and sole side streams. The authors used a specific energy ranging from 123 to 300 kJ/kg and an electric field strength between 1 and 3 kV/cm to obtain the compounds. These parameters were also used by the authors to obtain extracts with anti-inflammatory activity and the ability to modulate the intestinal microbiota from the same side streams [10]. Lastly, Franco et al. [17] used PEF pre-treatment to extract antioxidant compounds from sea bream and sea bass side streams. They used 100 pulses, a frequency of 10 Hz, a pulse width of 20 µs, and a specific energy ranging from 17.4 to 29.4 kJ/kg. These studies demonstrate the potential of PEF technology for the recovery of valuable compounds from fish side streams.

As mentioned before, only one study has applied PEF for the valorization of sea bass side streams [17]. However, in this study, the authors only focused on the analysis of total antioxidant activity of the methanolic and aqueous extracts obtained from sea bass gills, bones, and heads assisted by PEF and conventional extraction. At this stage of development, characterizing the complete compound profile is crucial rather than solely focusing on total antioxidant capacity. Expanding information and data on recovering high-added-value compounds from sea bass side streams with PEF assistance is vital due to limited knowledge. Considering the presence of heavy metals, the primary contaminants in fish and fish-derived products is important. Additionally, supplementing information with nutritionally significant compounds, such as bioactive peptides or minerals, is necessary.

The present study aims to enhance understanding of extracts from sea bass side streams (head, skin, viscera, and backbone), focusing on a comprehensive analysis of their composition, including protein analysis, peptides identification, and mineral quantification. Investigating heavy metal presence is also essential for characterizing marine biomass, and, for that reason, this is also studied in the present work. Furthermore, the study examines the impact of extracts on cell viability in human cell lines. By expanding knowledge on recovering valuable compounds from sea bass side streams with PEF assistance, the study aims to fill knowledge gaps. Considering heavy metal presence and other nutritionally significant compounds contributes to a better understanding of potential applications and nutritional value.

## 2. Materials and Methods

### 2.1. Chemicals and Reagents

Sodium fluorescein was acquired from Fluka Chemie AG (Bunds, Switzerland). Coomassie brilliant blue R250, Precision Plus Protein™ 5–250 kDa (molecular weight marker), and 8–16% Mini-PROTEAN^®^ TGX™ Precast gels were bought from BioRad company (Hercules, CA, USA). Dithiothreitol (DTT) was purchased from VWR (Leuven, Belgium). In order to obtain highly pure water (resistivity >18 MΩ/cm), a Milli-Q SP Reagent Water System (Millipore Corporation, Bedford, MA, USA) was used. The K_2_S_2_O_8_, Trolox, ABTS, and AAPH reagents were procured by Sigma–Aldrich (Steinheim, Baden-Württemberg, Germany). Hydrochloric acid (HCl), and Sulfuric acid 96% (H_2_SO_4_) were purchased from Merck (Whitehouse Station, NJ, USA). Potassium phosphate monobasic (KH_2_PO_4_), boric acid (H_3_BO_3_), sodium phosphate dibasic (Na_2_HPO_4_), and potassium phosphate dibasic (K_2_HPO_4_), were purchased from Merck (Darmstadt, Germany). Lastly, ethanol 96% was bought from Panreac (Castellar del Vallés, Barcelona, Spain).

### 2.2. Samples

Sea bass (*Dicentrarchus labrax*) fresh deceased fish samples were obtained from a local market and were kept at 4 °C until the analysis. The different side streams (head, skin, viscera, and backbone) were separated manually and stored at −20 °C. The composition of each side stream has already been described by Munekata et al. [12].

### 2.3. Optimization Process

For the optimization study, a response-surface methodology was used by means of a Box-Behnken design with two central points. The studied parameters were specific energy (50–300 kJ/kg), field strength (1–3 kV/cm), and time of extraction (0–24 h). The specific energy range was selected based on previous preliminary experiments (data not shown). In addition, field strength range was selected based on the possibilities of the PEF machine, as it only allows testing in a range from 1 to 3 kV/cm. The responses analysed were: (i) total protein and (ii) antioxidant capacity measured with two methods, TEAC and ORAC. As shown in Table S1, 15 different experiments were set with the combination of the minimum, central, and maximum values of each parameter. Moreover, a replicate of the central point was performed to check the variability and the reproducibility. Statgraphics Centurion XVI^®^ (Statgraphics Technologies, Inc., The Plains, VA, USA) was used for the optimization process.

### 2.4. Pulsed Electric Fields (PEF) Treatment

For the PEF treatment of the different side streams, the PEF-Cellcrack III equipment (ELEA, Quakenbrück, Germany) available at the Faculty of Pharmacy of the University of Valencia was used. A 900 mL treatment chamber and a sample:water ratio of 1:15 were chosen. The specific energy (kJ/kg) and field strength (kV/cm) parameters were set according to the experimental design (Table S1). The pulse duration and frequency remained constant at 100 ms and 2 Hz, respectively. The pulse form was unipolar square wave pulse. The conductivity and temperature of the sample were measured before and after the treatment using a portable conductivity meter ProfiLine Cond 3310 (WTW, Xylem Analytics, Weilheim in Oberbayern, Germany). Despite a significant increase in temperature when using a high specific energy (e.g., 300 kJ/kg for skin optimal extract), its influence was minimized when comparing the control extraction and the sample pre-treated with PEF, since both extractions were carried out at the same temperature. Therefore, the only factor that was different was the pre-treatment PEF, and only changes due to this treatment were observed, which was the aim of the study.

### 2.5. Supplementary Aqueous Extraction

After the PEF treatment, samples were stirred at 200 rpm from 0.5 to 24 h, according to the experimental design (Table S1). Samples were then centrifuged at 3050× *g* for 10 min in a 5810 R centrifuge (Eppendorf AG, Hamburg, Germany). The supernatant was collected and kept frozen at −20 °C for further analysis.

### 2.6. Minerals and Heavy Metals Determination and Quantification

The mineral composition (Ca, Mg, P, Fe, Se, and Zn) and heavy metals (As, Pb, Hg and Cd) of each extract were analyzed according to the methods described by de la Fuente et al. [18]. Briefly, samples were digested in a microwave oven with H_2_O_2_ and HNO_3_ and were then filtered. Finally, the liquid fraction was injected in an inductively coupled plasma spectrometer mass detector (ICP-MS model 7900, Agilent Technologies, Santa Clara, CA, USA) for the detection and quantification of the minerals and heavy metals.

### 2.7. Proteins

#### 2.7.1. Total Protein Content

The total protein content of the obtained extracts was determined as described by Al Khawli et al. [14].

#### 2.7.2. Molecular Weight Distribution

For the analysis of molecular weight distribution, an SDS-PAGE electrophoresis was performed based on the method previously described by de la Fuente et al. [15]. First, proteins were precipitated with cold acetone (ratio sample:acetone 1:4 (*v*/*v*)). Then, samples were centrifuged at 9000× *g*, the supernatant was removed, and the pellet was resuspended in deionized water. Sample buffer (62.5 mmol/L Tris-HCl (pH 6.8), 2 g/100 g SDS, 20 g/100 g glycerol, 0.01 g/100 g bromophenol blue, and 50 mmol/L dithiothreitol) was added to that samples and they were denaturalized at 95 °C for 5 min. Subsequently, 10 µL of the mixture were loaded on an 8–16% Mini-PROTEAN^®^ TGX™ Precast gel and the electrophoresis was run 30 min at 120 V and then at 80 V until the end. Glycine (192 mmol/L), Trizma^®^ base (25 mmol/L) and SDS (0.1 g/100 g) were mixed to prepare the running buffer. Precision Plus Protein™ 5–250 kDa was used in order to estimate the molecular weight of the bands. Once electrophoresis was finished, 0.125% Coomassie brilliant blue R–250 was used to stain the gel. Subsequently, a mixture of acetic acid (10 g/100 g) and methanol (20 g/100 g) (in water) was utilized to distain the gel.

#### 2.7.3. Bioactive Peptides Identification

Bioactive peptide identification was carried out according to the method proposed by de la Fuente et al. [19]. Once the soluble peptides were isolated, they were analyzed using a nanoESI qTOF mass spectrometer (6600plus TripleTOF, ABSCIEX, Framingham, MA, USA), equipped with a trap column (ChromXP C18, 3 μm 120 Å, 350 μm, 0.5 mm; Eksigent). After the LC-MS/MS was performed, the identification of the different peptides was conducted using the software ProteinPilot v5.0 search engine (AB SCIEX). Finally, the bioactivity and potential bioactivity of the different peptides were checked using the BIOPEP-UWM database [20].

### 2.8. Total Antioxidant Capacity Determination

In order to determine Total Antioxidant Capacity (TAC), both Trolox Equivalent Antioxidant Capacity (TEAC) and Oxygen Radical Absorbance Capacity (ORAC) assays were used. Regarding TEAC, 440 µL of K_2_S_2_O_8_ 140 mmol/L were added to 25 mL of ABTS 7 mmol/L and kept in darkness at room temperature for 12–16 h to obtain ABTS•+ radical. The solution was diluted with ethanol until an absorbance of 0.700 ± 0.020 was reached at 734 nm, which was considered the initial absorbance. Next, appropriately diluted extracts (100 μL) were mixed with 2 mL of ABTS•+ radical, and the absorbance was measured after 3 min using a Perkin-Elmer UV/Vis Lambda 2 spectrophotometer (Perkin-Elmer, Jügesheim, Germany) with triplicate measurements. To determine the antioxidant activity, a standard curve was prepared using Trolox, and the percentage of inhibition (% Inhibition) was calculated for each sample as described in Equation (1), with A_f_ being the absorbance after 3 min and A_0_ being the initial absorbance. The percentage of inhibition was then interpolated to determine the antioxidant activity, expressed as µmol trolox equivalent/L extract (µmol TE/L) (Equation (1)).

% Inhibition = (1 − (A_f_/A_0_)) × 100 (1)

The ORAC assay was employed to assess antioxidant capacity based on the ability to eliminate peroxyl radicals, using the method outlined by de la Fuente et al. [18].

### 2.9. Cell Cultures and Assessment of Cell Viability

#### 2.9.1. Cell Culture

The human neuroblastoma SH-SY5Y cell culture was carried out according to Zingales et al. [21]. Cells were cultured in DMEM Ham’s-F12 medium supplemented with 10% fetal bovine serum (FBS), 100 U/mL penicillin, and 100 mg/mL streptomycin. The cells were incubated under specific conditions: pH 7.4, 5% CO_2_ at 37 °C, and 95% air atmosphere with constant humidity. The culture medium was changed every 2–3 days. Different concentrations of fish side streams extracts (head, skin, viscera, and backbone) were tested. Extract concentrations tested were 1:2 dilutions (25% to 0.78%). Control groups were included in each experiment.

#### 2.9.2. Assessment of Cell Viability

In order to determine cell viability of sea bass side streams extracts (head, skin, viscera, and backbone) obtained by PEF and by agitation (control), the MTT assay in SH-SY5Y cells was performed following the method described by Zingales et al. [21]. Briefly, 30,000 cells/well were seeded in 96-well plates for 48 h until they reached 80% of confluence. The culture medium was then removed and cells were exposed individually to sea bass side streams extracts at increasing concentrations from 0.78 up to 25%, obtained by both methods carried out, for 24 h. After the incubation period, the culture medium containing the extract was replaced with fresh medium containing 50 μL of MTT salt (5 mg/mL PBS). After 3 h of incubation at 37 °C in the dark, the resulting formazan crystals were dissolved in DMSO. An automatic plate reader (MultiSkanEX, Labsystem, Helsinki, Finland) was utilized to measure absorbance at 540 nm. Cell viability was expressed as a percentage relative to the control.

### 2.10. Statistical Analysis

Significant differences between the results were determined by performing a t-test or an analysis of variance (ANOVA). In addition, the least significant differences (LSD) test was used. A *p <* 0.05 was considered significant. The software GraphPad Prism 8 (GraphPad Software, Inc., San Diego, CA, USA) was used for the statistical analysis.

## 3. Results

### 3.1. Optimization Process and Comparison with Control Sample

The obtained results for each response (total proteins, TEAC, and ORAC) and each side stream are shown in the Supplementary Material (Tables S2–S5 and Figures S1–S4). As expected, the increment of temperature and conductivity due to the PEF treatment was greater as the electric field strength and the specific energy increased. As expected, temperature and conductivity increase due to the PEF treatment was greater as the electric field strength and the specific energy increased. The increase in conductivity is correlated with a higher release of intracellular compounds due to the PEF effect. Furthermore, as mentioned earlier, the impact of temperature increase was minimized by conducting the control at the same temperature achieved under optimal conditions. This ensured that the only differing factor was the PEF treatment.

The optimization was carried out in order to maximize the value of each studied response. The optimal conditions for each side stream are presented in Table 1.

**Figure 1:**
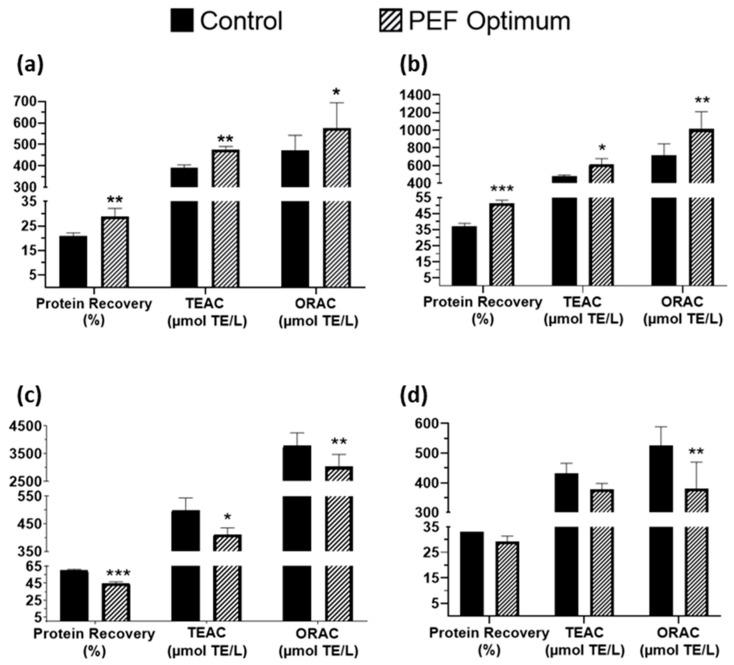
Comparison between PEF-pretreatment at optimal conditions and control sample at the same time of extraction for the studied side streams: (**a**) head, (**b**) skin, (**c**) viscera, and (**d**) backbone. Results are expressed as mean ± standard deviation (SD). * = *p* < 0.05; ** = *p* < 0.01; *** = *p* < 0.001.

Then, the extractions were performed following the abovementioned optimal conditions. The obtained results were compared to those obtained after the control process (without PEF pre-treatment) (Figure 1). As can be observed in Figure 1, the PEF pre-treatment increased protein recovery for the head (from 21.01 ± 1.14 (control) up to 28.92 ± 3.22% (PEF Optimum) of total proteins) and the skin (from 37.25 ± 1.63 up to 51.34 ± 1.98% of total proteins) extracts, while it had a negative effect on viscera extracts, decreasing the recovery compared to the control. Regarding the antioxidant activity, behavior was similar for both TEAC and ORAC assays, with an increase in the antioxidant capacity values of head and skin extracts obtained by PEF observed, and with decreased antioxidant values for the viscera extracts compared to the control samples. For the TEAC assay, the values for head and skin improved by 21.74 and 29.11%, respectively, while for viscera, they decreased by 17.65%. In addition, ORAC results increased by 22.11% (head) and 40.93% (skin), while for viscera extracts, the value was reduced by 19.88%. Finally, the backbone extracts did not show any significant differences in total protein or TEAC values after the PEF compared to the control, while ORAC values were higher in the control samples than in the PEF extract (526.38 vs. 379.29 µM TE, respectively). The improvement in protein and antioxidant compounds extraction from the head and skin can be explained by the electroporation process promoted by PEF processing. In general, PEF affects biological cells and leads to specific structural changes and destruction of the cell membrane, and then it helps release intracellular components, thus increasing the extraction yield of different compounds, such as protein or antioxidant compounds. However, in the case of viscera extracts, no improvement was observed compared to the control samples. This can be explained because the electric field strength used was 3 kV/cm, and it has been observed by other authors up to 10 kV/cm that the effect is not significant. Electric fields of at least 10 kV/cm are required to electroporate fish viscera, with the optimal one being 20 kV/cm [22]. Therefore, the intensity used in our study is lower than the required amount to electroporate and promote an improvement in the extraction yield.

These results do not agree with the data showed by Franco et al. [17], who did not detect any significant difference in antioxidant capacity measured by the TEAC method for sea bass head and bone extracts after PEF treatment. However, this can be explained by the method of preparation of the sample. In our study, the sample was treated directly, putting the side stream directly in the treatment chamber, while in the study of Franco et al., the sample was intensively crushed and vortexed. This step can hide the effect of PEF, as the cells are not intact, and then the intracellular components are already released after the crushing process. On the other hand, our results fully agree with the data reported by Wang et al. [9], who obtained a higher protein recovery in sole head and skin extracts after PEF pre-treatment, while no significant differences were observed for viscera. Moreover, the highest protein recovery was also obtained with skin extract, as in our study.

### 3.2. Distribution of Protein Molecular Weight

**Figure 2:**
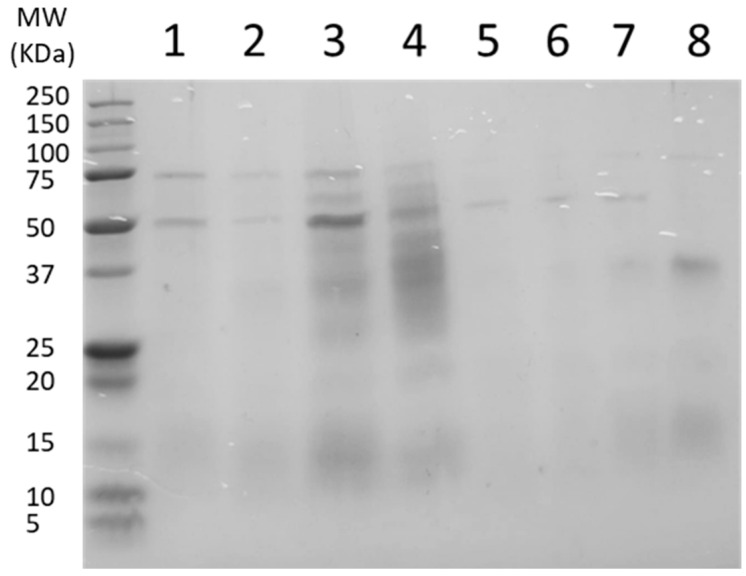
SDS-PAGE gel of the side streams extracts. 1: Head with pulsed electric fields (PEF) pre-treatment; 2: Head control; 3: Skin with PEF pre-treatment; 4: Skin control; 5: Viscera with PEF pre-treatment; 6: Viscera control; 7: Backbone with PEF pre-treatment; 8: Backbone control.

The molecular weight of the control and the PEF extracts’ protein profile was analyzed. Figure 2 depicts the SDS-PAGE of the various samples. Notably, this method also offers insight into the overall protein content. As can be seen, skin extracts had the highest amount and variety of proteins. Moreover, the ability of PEF to preserve high molecular weight proteins is also remarkable. In this sense, it can be observed that for the PEF pre-treated backbone extract, there is a band at 50 kDa that is lost in the backbone control extract. This effect can also be observed for skin samples, in which the PEF sample has a more intense band at 50 kDa, while in the control sample, it seems that this band has been degraded, showing a more intense color in a range from 25 to 37 kDa.

These results also show that skin extracts had the highest protein content, while viscera extracts had the lowest values. However, this gel does not allow peptides to be retained and studied, so they have been analyzed qualitatively using a nanoESI qTOF mass spectrometer, and this will be discussed in the next section.

### 3.3. Bioactive Peptides Identification

The presence of peptides with antioxidant or potential antioxidant activity in each extract was studied. In the backbone extract obtained after PEF treatment, the peptide LEQQVDDLEGSLEQEKK was found, which has antioxidant activity, as previously demonstrated by Je et al. [23]. Moreover, other potential antioxidant peptides were found, which are presented in Table 2 (only peptides with more than 90% of confidence were selected). The BIOPEP-UMP database did not reveal any association between these peptides and antioxidant activity. Nevertheless, a bioinformatic analysis revealed the presence of several short amino acid sequences with antioxidant properties within these peptides, as shown in Table 2.

The potential antioxidant activity of the peptides evaluated was based on the presence of specific amino acids in the peptide sequence [24]. It is known that amino acids with aromatic rings in their molecule have good antioxidant capacity. These amino acids are tryptophan (W), tyrosine (Y), and phenylalanine (F), which are aromatic amino acids associated with a higher antioxidant capacity due to the presence of an aromatic ring and their ability to transfer protons and/or electrons. Moreover, histidine (H) is also associated with strong radical-scavenging activity due to its imidazole ring. In addition, the pyrrolidine and indole rings of proline (P) and W are able to act as hydrogen donors due to the presence of hydroxyl groups, being hydroxyl radical scavengers [24]. As can be seen in Table 2, PEF pre-treatment can affect the variability of the peptides. For head extracts, PEF increases the diversity of peptides, resulting in a wider range of peptides found in the pre-treated sample compared to the control sample. However, for skin extracts, the effect is the opposite: PEF reduces the variety of peptides compared to the control sample. It should be noted that the highest amount of potential antioxidant peptides was found in backbone samples, probably due to the presence of remnants of fish flesh adhering to the backbone. Finally, it should be mentioned that the analysis of potential bioactive peptides was just qualitative, not quantitative. It cannot be correlated with the different amounts of obtained peptides with antioxidant capacity, then, as a different proportion of each peptide can be found in each extract.

Regarding the effect of the treatment on peptides, it has been seen that PEF treatment can modify the secondary structure of peptides, mainly in the alpha-helix content. However, the relationship between antioxidant capacity and these changes is not clear and needs further study. On the other hand, it has also been seen that PEF treatment can alter the spatial conformation of the molecule, exposing aromatic amino acids and thus modifying the antioxidant capacity of the peptide [25]. This alteration in conformation could explain the observed higher antioxidant capacity in PEF extracts of the fish head and skin compared to the control samples. However, the lower antioxidant capacity observed in the PEF extract of the fish viscera, in comparison to the control sample, requires further investigation to understand the underlying factors involved.

With respect to the peptides obtained, the peptide IITNWDDMEKIWHHT, which contains three sequences with antioxidant capacity (HH; WHH; WDDMEK), is noteworthy. The SEALKDAQEKLELAE sequence also has a large presence of amino acid combinations with antioxidant capacity, with four different groups (DAQEKLE; EL; KD; LK). It can also be observed that LLIVYPWTQR presents five different groups with potential antioxidant activity (PWT; PW; VY; YPW; YPWT). Finally, the sequence SADTLWGIQKDLKDLKDL has up to five groups with antioxidant potential (LWG; KD; LK; LW; WG), too.

Other authors also obtained a significant influence when using non-conventional technologies to obtain bioactive peptides from fish side streams. In this sense, de la Fuente et al. [19] obtained 137 potential antioxidant peptides after the application of pressurized liquid extraction (PLE) technology on salmon viscera. However, these authors only obtained 67 potential antioxidant peptides when using shaking extraction (control). These results confirm that the technology used for the extraction procedure affects the obtained products, allowing the recovery of different peptides and other bioactive compounds. In addition, after the different procedures, the sequences GAA, GPP, and EL were found in the antioxidant extracts.

In addition, the same antioxidant peptide sequences after the treatment of different animal foods with different methods were described in other studies. In this sense, Saiga et al. [26] reported the presence of the peptides DAQEKLE, IEAEGE, DSGVT, VPSIDDQEELM, and EELDNALN in the extracts obtained after the enzymatic treatment of pork muscles, with the sequence DAQEKLE being the one with the highest antioxidant capacity. Zielińska et al. [27] found the sequence AGDDAPR after the in vitro digestion of *Tenebrio molitor*. Finally, Oliveira Lima et al. [28] described the presence of the peptide sequence IITNWDDMEK, with the antioxidant group WDDMEK, in hydrolyzed samples from stripped weakfish (*Cynoscion guatucupa*) by-products by enzymatic hydrolysis using Protamex.

### 3.4. Mineral Content

**Figure 3:**
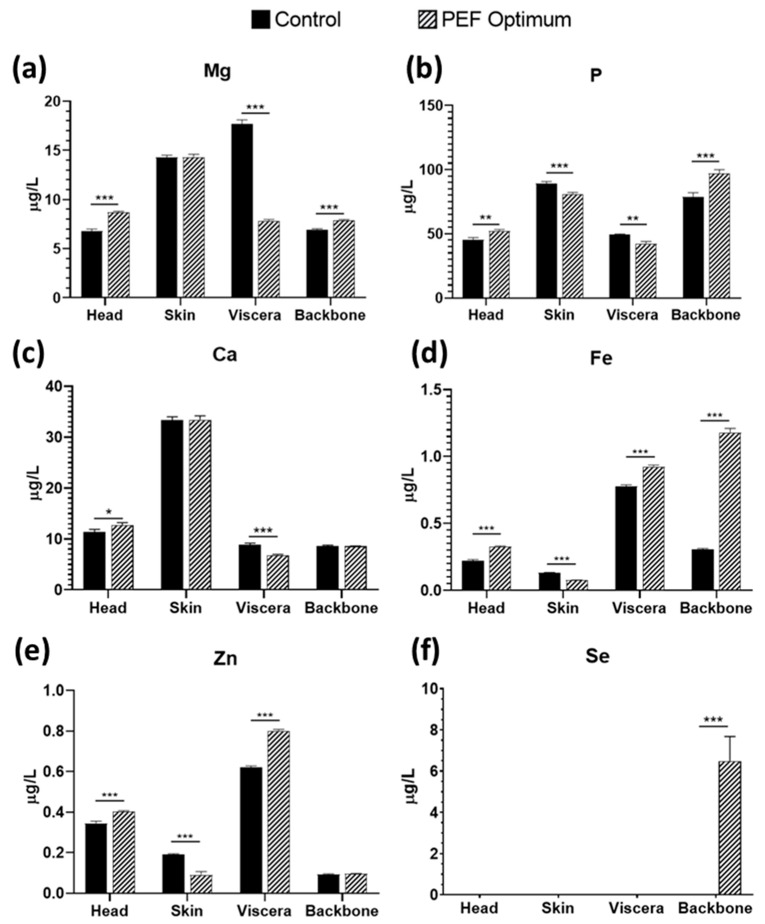
Concentration of: (**a**) Mg, (**b**) P, (**c**) Ca, (**d**) Fe, (**e**) Zn, and (**f**) Se in sea bass side stream aqueous extracts, comparing control extraction by soaking vs. PEF pre-treatment. * = *p* < 0.05; ** = *p* < 0.01; *** = *p* < 0.001.

Regarding mineral content, Mg, P, Ca, Fe, Zn, and Se were analyzed by ICP-MS (Figure 3). For head side stream, PEF increased the extraction of Mg, P, Ca, Fe, and Zn, while for skin, PEF pretreatment had only a significant effect for P, Fe, and Zn. On the other hand, for viscera, the highest concentration of Fe and Zn was observed for the PEF-treated extracts, while the control extracts presented a higher recovery of Mg, P, and Ca. Finally, PEF treatment had a significant effect on recovering Mg, P, and Fe in backbone extracts. It is worth mentioning that Se was only found in the PEF-pretreated backbone extracts. Although the application of PEF had different effects according to the targeted each mineral and side stream, it can, in general, be concluded that it is a promising technology to enhance mineral recovery.

Se intake is extremely variable across the world [29], and the health benefits still need to be defined [30]. However, it is known that is involved in the antioxidant mechanism of the cells through the enzyme GSH, which catalyze the reduction of hydrogen and lipid peroxides [31]. Se is also involved in the regulation of several antioxidant genes, such as superoxide dismutase (SOD) and catalase (CAT) [32]. Moreover, its synergic effect with vitamin E as antioxidants is also known, which protects the cell membrane [31]. In addition, Zn is also a relevant mineral regarding antioxidant function in the human body. As Se, it also contributes to the correct function of antioxidant enzymes (GSH and SOD) [33]. Moreover, Zn is also able to inhibit NADPH-oxidase, reducing the production of reactive oxygen and nitrogen species [34].

PEF technology allowed its recovery, and the extracts obtained could be used for the development of nutraceuticals. In most cases, PEF enhanced the extraction of Fe and Zn, and certain population intakes of these two minerals are provided by eating small fish whole [35]. However, in order to use the bones of large fish in food products, the bone structure must be softened. This can be achieved through various methods, such as the application of hot water, hot acetic acid, or steam. [36]. In this case, PEF technology is a green alternative that, with further processing, could provide similar results.

### 3.5. Heavy Metals Quantification

**Figure 4:**
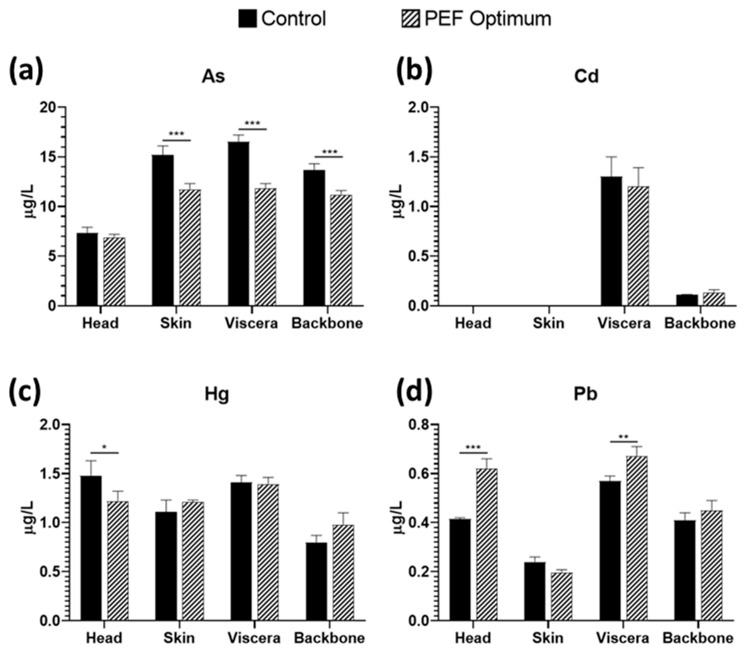
Concentration of (**a**) As, (**b**) Cd, (**c**) Hg, and (**d**) Pb in sea bass side streams aqueous extracts, comparing control extraction by soaking vs. PEF pre-treatment. * = *p* < 0.05; ** = *p* < 0.01; *** = *p* < 0.001.

Cd, Hg, and Pb were analyzed by ICP-MS, and are the main contaminants found in marine fish [37]. As can be observed in Figure 4, PEF pre-treatment had a significant effect on reducing the concentration of As of skin, viscera, and backbone extracts. Moreover, the PEF treatment also reduced the concentration of Hg in the head extract. On the other hand, the application of PEF increased the release of Pb to the extract in the head and viscera side streams. Finally, PEF did not have a significant effect on Cd concentrations.

The heavy metal concentration ranges varied from 6.9–16.5, 0–1.3, 0.8–1.48, and 0.197–0.67 µg/L of extract for As, Cd, Hg, and Pb, respectively. The most predominant heavy metal was found in the head, skin, and backbone samples, followed by Hg, Pb, and Cd. Conversely, for viscera samples, the order of toxic metal concentration was As > Hg > Cd > Pb, with a decreasing trend, and with the side stream having the highest Cd concentration (1.2–1.3 µg/L). All the values obtained are below the limits set by the EFSA for Cd, Hg, and Pb of 0.050, 0.5, and 0.30 mg/kg wet-weight muscle meat, respectively [38]. In the case of As, there is not a maximum limit set, but only recommendations about the daily intake. Nevertheless, the type of As (organic or inorganic) need to be elucidated because the organic forms present in some mollusks and crustaceans have not been shown to produce adverse effects in humans consuming this seafood [39].

### 3.6. Effect of Fish Side Stream Extracts on Cell Viability

**Figure 5:**
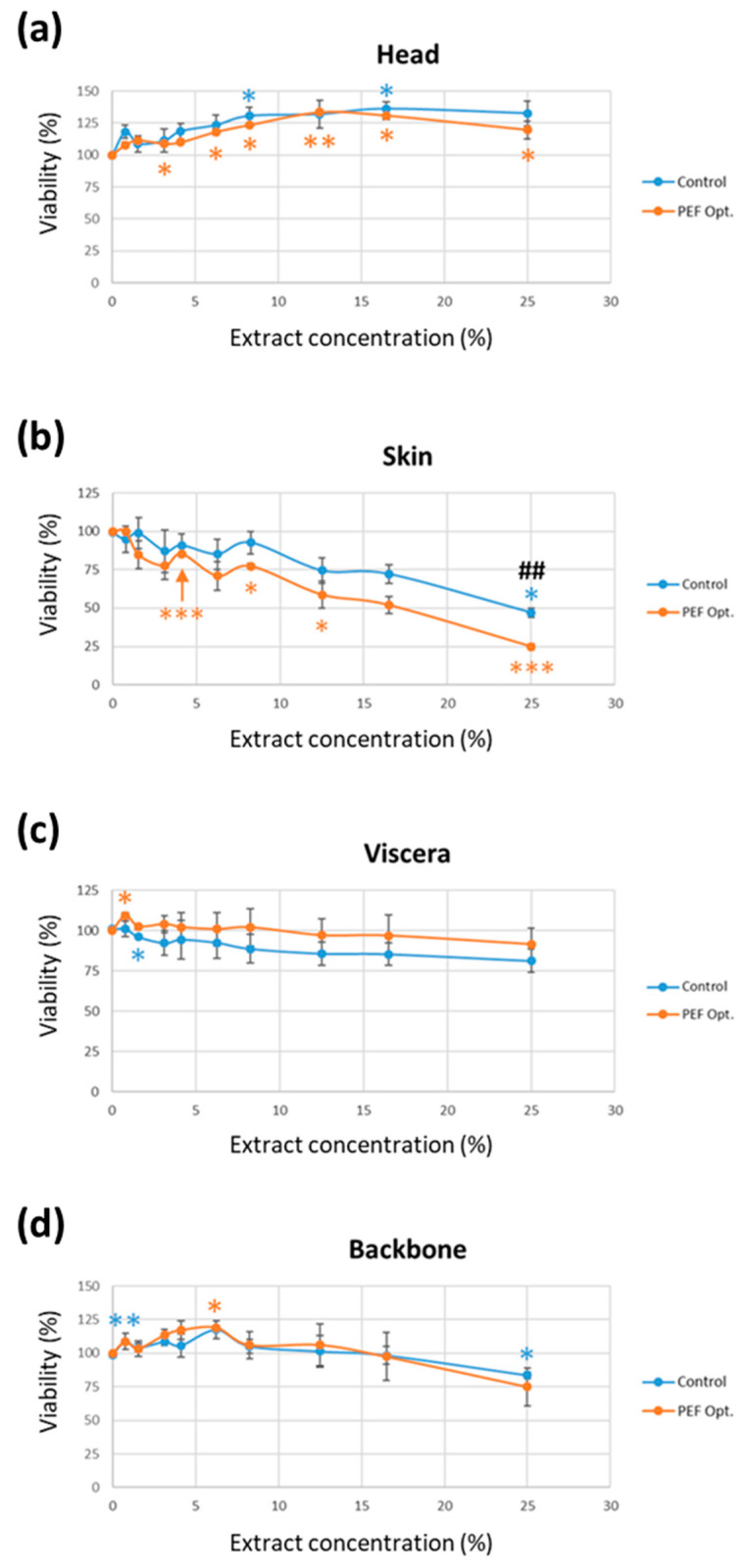
Effect of fish side streams extracts (**a**) head; (**b**) skin; (**c**) viscera and (**d**) backbone obtained by pulsed electric fields pre-treatment (“PEF Opt.”) and its control on cell viability after 24 h of exposure. Cell viability was measured by MTT assay on SH-SY5Y cells. The results are expressed as mean ± SEM of three independent experiments. * = *p* < 0.05 vs. its respective cell control (0% extract); ** = *p* < 0.01 vs. its respective cell control (0% extract): *** = *p* < 0.001 vs. its respective cell control (0% extract); ## = *p* < 0.01 between PEF and control extracts.

In order to determine the cell viability of the sea bass side streams extracts obtained by PEF and agitation (control) methods, a MTT assay in SH-SY5Y cells for 24 h was performed (Figure 5).

The results of the extract concentration-cell viability assay demonstrated that the highest cell viability (33% and 25%) was achieved with 12.5% and 8.5% head extract obtained by PEF and agitation (control), respectively (Figure 5a). However, both skin extracts, the one obtained by PEF and the one obtained by agitation (the control in Figure 5b), showed a significant decrease in cell viability compared to cells not exposed to any extract (0% extract). Moreover, SH-SY5Y cell exposed to PEF skin extracts resulted in a major cell viability decrease compared to the agitation method (control in Figure 5b). In contrast, the PEF and agitation (control) extracts of backbone and viscera did not exhibit any significant changes when compared to each other. Nevertheless, the 6.25% backbone PEF extract exhibited a significant increase in cell viability at 6.25% concentration with respect to the cell (0% extract).

Our results are similar to the data reported by other authors. The hydrolysates derived from fish by-products are the primary focus of study in the literature due to their potential as valuable products with diverse bioactive properties. In this sense, in a recent work, Taroncher et al. [40] studied the effects of fish hydrolysates in Caco-2/TC7 cells viability. They examined different by-products of salmon (S), mackerel (M), and herring (H): heads (HSH), backbones (HSB, HMB), and viscera (HSV, HMV, HHV). Within the tested concentrations (0.03125–1 mg/mL), hydrolysates had minimal impact on cell viability by MTT assay, except for HSB (0.125 mg/mL) and HSV (0.0625 mg/mL and 0.25 mg/mL), which showed significant cell viability increases (27% for HSB and 51.2% for HSV). No cytotoxic effects were observed by these authors. Respecting the total protein content (PC) assay, Taroncher et al. found that all hydrolysates, except HSB and HMV, increased cell viability. Notable increases in PC were observed for HMB (18%), HSV (19%), HHV (139%), HSH (140%), and HMH (214%). These results indicated that hydrolysate exposure enhanced cell viability in Caco-2/TC7 cells. Compared to our results, similar findings were obtained for heads and backbones, while Taroncher et al. obtained higher cell viability after exposure to viscera hydrolysates.

The cytoprotective effect of other marine by-products has been previously reported. The research conducted by Zhong et al. [41] explores the protective effect of hydrolysates derived from silver carp byproducts against oxidative stress. These authors demonstrated a substantial radical-neutralizing capacity of these hydrolysates on Caco-2 cells exposed to low concentrations of H_2_O_2_. These findings are consistent with the observations made by Hu et al. [42], who evaluated the oxidative stress in HepG2 cells exposed to hydrolysates obtained from monkfish (*Lophius litulon*) muscle. The study revealed a cytoprotective effect, leading to increased cell viability. Furthermore, these authors reported no cytotoxic effects associated with the studied products. Similarly, Gómez et al. [43] observed a dose-dependent increase in cytoprotective effect in HepG2 cells exposed to hydrolysates derived from side streams of red tilapia for 24 h.

Moreover, it has been observed that other hydrolysates derived from marine biomass are also non-cytotoxic. Wiriyaphan et al. [44] reported no cytotoxic effects of the hydrolysate derived from *Nemipterus* spp. side streams in Caco-2 cells. Zheng et al. [45] also demonstrated that human umbilical vein endothelial (HUVECs) cell viability did not decrease when they are exposed to hydrolysate obtained from swim bladders of *Nibea japonica* for 24 h.

## 4. Conclusions

In conclusion, PEF treatment increased the extraction of proteins and altered the molecular size distribution in sea bass side streams. Moreover, it enhanced the antioxidant activity and generation of bioactive peptides in the skin and head extracts. PEF also improved the recovery of essential minerals, such as Fe, Zn, and Mg, while reducing the presence of heavy metals. Additionally, head extracts exhibited improved cell viability compared to the control samples. However, the application of PEF in treating marine side streams is limited due to the high financial investment required for the equipment. Nevertheless, the advantages of short processing time and low energy demand make PEF technology a promising option for valorizing side streams and recovering valuable compounds from marine waste. This technology thus shows promise for the marine food industry and sustainability efforts by effectively utilizing fish waste, reducing environmental impact, and creating economic opportunities.

## Figures and Tables

**Table 1:** Optimal conditions regarding protein recovery, TEAC, and ORAC based on the studied side streams.

Side Stream	Specific Energy (kJ/kg)	Field Strength (kV/cm)	Time of Extraction (h)
Head	220	1	21.35
Skin	300	3	24
Viscera	123.72	3	15.17
Backbone	300	1	21

**Table 2:** Peptides found in the different extracts with potential antioxidant capacity and the group responsible for this bioactivity. Pulsed electric fields (PEF) (only peptides with more than 90% of confidence provided by BIOPEP-UMP database were selected).

	Sequence	Involved Amino Acids
HEAD CONTROL	ATDGGAHGVINVSVSEAAIEASTR	AH; GAH
	IIDQDKSGFIEEDELKL	EL; LK
	IIDQDKSGFIEEDELKLFLQN	EL; LK
	MEHDPQARDRKAQEL	EL
	SANLMAGHWVAISGAAGGLGSLAVQYAK	GAA
HEAD PEF OPT	AISEELDHALNDMTSI	EL
	AVRNDEELNKLLGGVTI	EL
	AVRNDEELNKLLGGVTIAQGGVLPNIQA	EL
	AVRNDEELNKLLGGVTIAQGGVLPNIQAVLLPK	EL
	ENKNLQQEISDLTEQLGETGKSIHELEK	EL
	GTEDELDKYSEALKDAQEKLELAEKKATDAEGD-VAS	DAQEKLE; EL; KD; LK
	GTEDELDKYSEALKDAQEKLELAEKKATDAEGD-VASLNR	DAQEKLE; EL; KD; LK
	ISEELDHALNDMTSI	EL
	LLIVYPWTQR	PWT; PW; VY; YPW; YPWT
	MSADAMLKALLGSK	LK
	NDEELNKLLGGVT	EL
	NDEELNKLLGGVTIAQGGVLPNIQAVLLPK	EL
	NLQQEISDLTEQLGETGKSIHELEK	EL
	VAKLEKTIDDLEDELYAQK	LY; EL
	VFLENVIRDAVTYT	IR; TY; VFL
	VFLENVIRDAVTYTEHAK	IR; TY; VFL
	VFLENVIRDAVTYTEHAKR	IR; TY; VFL
	VGAGAPVYLAAVLEYLTAEILELAGNAAR	EL; VY
	VLEYLTAEILELAGNAARDNKKT	EL
	YKAISEELDHALNDMTSI	EL; KAI
SKIN CONTROL	AFGLFDRVGDNKVAYNQ	AY
	AGLLTSRSPTGSLWVVTA	LWV; LW
	AVGKVIPELNGKITGMA	EL; KVI; PEL
	AVGKVIPELDGKLTGMA	EL; KVI; PEL
	AVGKVIPELNGKITG	EL; KVI; PEL
	AVGKVIPELNGKLTG	EL; KVI; PEL
	AVGKVIPELNGKLTGMA	EL; KVI; PEL
	FAGDDAPRAVFPS	AGDDAPR
	FGLFDRVGDNKVAY	AY
	IIPASTGAAKAVGKVIPELNGK	EL; KVI; PEL; GAA
	IIPASTGAAKAVGKVIPELNGKITGMA	EL; KVI; PEL; GAA
	IIPASTGAAKAVGKVIPELNGKLTGMA	EL; KVI; PEL; GAA
	LRVFDKEGNGTVMGAELR	EL
	SSSLEKSYELPDGQVITIGNER	EL
	SVAELGEQIDNLQR	EL
	SVAELGEQIDNLQRVKQKLEKEKSE	EL
	SYELPDGQVITIGNER	EL
	TQQLEDLKRQLEEEVKAKN	LK
	TQQLEDLKRQLEEEVKAKNALAH	AH; LK
	VAELGEQIDNLQRVKQKLEKEKSE	EL
	VGKVIPELNGKITGMA	EL; KVI; PEL
	VGKVIPELNGKLTGMA	EL; KVI; PEL
	VLSGGTTMYPGIADRM	MY
	VLSGGTTMYPGIADRMQKE	MY
	VLSGGTTMYPGIADRMQKEITA	MY
SKIN PEF OPT	AFGLFDRVGDNKVAYNQIADIMR	AY
	DDEETTALVCDNGSGLVKAGFAGDDAPRA	AGDDAPR
	GAAAGAGAGAAGAGAAAGAEGPAGGPTGGP	GAA
	GKKMFGKQAGEDESDDFAIGGSTPTNKLK	LK
	GPAGAGAGDEAVDGATLYVKNLSF	LY
	IITNWDDMEKIWH	WDDMEK
	IITNWDDMEKIWHHT	HH; WHH; WDDMEK
	PIEHGIITNWDDMEKIWHHT	HH; WHH; WDDMEK
	QAGAAGGQPGAKAGGDDDVVDA	GAA
	VLSGGTTMYPGIADRMQKEITAL	MY
	YPIEHGIITNWDDMEKIWHHT	HH; WHH; WDDMK
VISCERA CONTROL	AIGLPDELIQK	EL
	AWGPGLEGGVVGK	AW; WG
	EVHLGWAAKGLGRKIQAMM	HL; MM
	FGGEHIPNSPF	GGE
	NIPTSGAEIGGAFGGEK	GGE
VISCERA PEF OPT	AIGLPDELIQK	EL
	AIIDQDKSGFIEEDEL	EL
	AIIDQDKSGFIEEDELK	EL; LK
	AWGPGLEGGVVGK	AW; WG
	GIELPYQDPAIK	EL
	GKDLFVSDLK	KD; LK; LFV
	IIDQDKSGFIEEDELK	EL; LK
	ILDQDKSGFIEEDELQ	EL
	IVNGEEAVPHSWPW	PHS; PW
	KEADAMAVDGGQVY	VY
	KVMGFVGIQTGFR	GFVG
BACKBONE CONTROL	AAVPSGASTGVHEALELR	EL
	AAVPSGASTGVHEALELRDGDKSRY	EL; RY
	AAVPSGASTGVHEALELRDGDKSRYLG	EL; RY; RYL; RYLG; YLG
	AAVPSGASTGVHEALELRDGDKSRYLGKGT	EL; RY; RYL; RYLG; YLG
	AAVPSGASTGVHEALELRDNDKANY	EL
	AGTNGETTTQGLDGLYER	LY
	AHQQTLDDLQAEEDKVNT	AH
	AKAVGKVIPELNGKLTGMA	EL; KVI; PEL
	AKYGKDATNVGDEGGF	KD
	AVGKVIPELNGKLT	EL; KVI; PEL
	AVGKVIPELNGKLTG	EL; KVI; PEL
	AVGKVIPELNGKLTGMA	EL; KVI; PEL
	AVPSGASTGVHEALELRDGDKSRY	EL; RY
	AVPSGASTGVHEALELRDGDKSRYLGKGT	EL; RY; RYL; RYLG; YLG
	AVRNDEELNKLLGGVTIAQGGVLPN	EL
	EEALDHLETLKRENKNLQQEISDLTEQLGETGKSI-HELEKA	HL; EL; LK
	EHGIVNNWDDMEKIWHHT	HH; WHH; WDDMEK
	EKTIDDLEDELYAQK	LY; EL
	ELPDGQVITIGNER	EL
	FDMFDTDGGGDISTKELGT	EL
	FMLELDGTENKSK	EL
	GIITNWDDMEK	WDDMEK
	GPMKGILGYTEHQ	LGY
	HIADLAGHKDVILP	KD
	IADLAGNEDVILPVPAFNVINGGSHAGNK	GSH
	IITNWDDMEKIWH	WDDMEK
	IITNWDDMEKIWHHT	HH; WHH; WDDMEK
	ISDLTEQLGETGKSIHELE	EL
	ISDLTEQLGETGKSIHELEK	EL
	ISDLTEQLGETGKSIHELEKA	EL
	ITATQKTVDGPSGKLWR	LWR; LW
	IVNNWDDMEKIWH	WDDMEK
	KLEKTIDDLEDELY	LY; EL
	KYGKDATNVGDEGGF	KD
	LAGTNGETTTQGLDGLYER	LY
	LAVRNDEELNKLLGGVTIAQGGVLPN	EL
	LEKTIDDLEDELY	LY; EL
	LPDGKVITIGNER	KVI
	LQLAVRNDEELNKLLGGVTIAQGGVLPN	EL; LQL
	LTEQLGETGKSIHELEK	EL
	LTGCTDIQIVGDDLTVTNPKR	TGC
	LTKLEEAEKAADESERGMKVIENR	KVI
	MLELDGTENKSKFGANA	EL
	MLELDGTENKSKFGANAILGVS	EL
	QGGGGWGGGPGGGQQGGGAP	WG
	SEALKDAQEKLELAE	DAQEKLE; EL; KD; LK
	SEALKDAQEKLELAEK	DAQEKLE; EL; KD; LK
	SEALKDAQEKLELAEKKATDAEGDVASLNR	DAQEKLE; EL; KD; LK
	SEALKDAQEKLELAEKKATDAEGDVASLNRR	DAQEKLE; EL; KD; LK
	SELKKDIDDLELTL	EL; KD; LK
	SELKKDIDDLELTLAK	EL; KD; LK
	SELKKDIDDLELTLAKVEKE	EL; KD; LK
	SELKKDIDDLELTLAKVEKEKHATEN	EL; KD; LK
	SELKKDIDDLELTLAKVEKEKHATENK	EL; KD; LK
	SKQLEDDLVALQKKLKGTEDELDKYSE	EL; LK
	SVAELGEQIDNLQR	EL
	SYELPDGQVITIGNER	EL
	TEQLGETGKSIHELEKA	EL
	VAKLEKTIDDLEDELY	LY; EL
	VEEELDRAQERLATALTKLEEAEKAADESERG	EL
	VEEELDRAQERLATALTKLEEAEKAADESERGMK	EL
	VEEELDRAQERLATALTKLEEAEKAADESERGMKVIENR	EL; KVI
	VGKVIPELNGKLTGMA	EL; KVI; PEL
	VINGGSHAGNKLAMQEFM	GSH
	VLSGGTTMYPGIADR	MY
	VVESTGVFTTIEKASAH	AH
	VVESTGVFTTIEKASAHIKGGAKR	AH
	YELPDGQVITIGNER	EL
BACKBONE PEF OPT	AALTGGAAAGVAGAAAAGPAGDIA	GAA
	AHQQTLDDLQAEEDKVNT	AH
	AISEELDHALNDMTS	EL
	AVRNDEELNKLLGGVTIAQGGVLPN	EL
	DAQEKLELAEKKATDAEGDVAS	DAQEKLE; EL
	EKTIDDLEDELYAQK	LY; EL
	FMIELDGTENK	EL
	GIITNWDDMEK	WDDMEK
	GPAGAGAGDEAVDGATLYVKNLSF	LY
	GTEDELDKYSE	EL
	GTEDELDKYSEALKDAQEKLE	DAQEKLE; EL; KD; LK
	HLQLAVRNDEELNKLLGGVTIAQGGVLPN	HL; EL; LQL
	IIDQDKSGFIEEDELKL	EL; LK
	IITNWDDMEK	WDDMEK
	IITNWDDMEKIWHHT	HH; WHH; WDDMEK
	ILDQDKSGFIEEDELQLFLQN	EL; LQL
	ISDLTEQLGETGKSIHELEK	EL
	ISEELDHALNDMTS	EL
	ISEELDHALNDMTSI	EL
	KKQADSVAELGEQIDNLQR	EL
	KLKGTEDELDKYSEALKDAQEKLELAEKKATDAEGDVASLNR	DAQEKLE; EL; KD; LK
	LAVRNDEELNKLLGGVTIAQGGVLPN	EL
	LEKTIDDLEDELYAQK	LY; EL
	LTKLEEAEKAADESERGMKVIENR	KVI
	MSADAMLKALLGSK	LK
	NLQQEISDLTEQLGETGKSIHELEK	EL
	PGPNKGDSRGPPNHHMGP	HH; NHH; GPP
	PGSPAGAATSAPGAPAPG	GAA
	PIEHGIITNWDDMEK	WDDMEK
	RIQLVEEELDRAQERLATA	EL
	SADTLWGIQKDLKDL	LWG; KD; LK; LW; WG
	SEALKDAQEKLELAEKKATDAEGDVAS	DAQEKLE; EL; KD; LK
	SEALKDAQEKLELAEKKATDAEGDVASLNR	DAQEKLE; EL; KD; LK
	SEALKDAQEKLELAEKKATDAEGDVASLNRR	DAQEKLE; EL; KD; LK
	SKQLEDDLVALQKKLKGTEDELDKYSEALKDAQEKLELAEKKATDAEGDVASLNR	DAQEKLE; EL; KD; LK
	SQKEDKYEEEIKVLTDKLK	LK
	SQKEDKYEEEIKVLTDKLKEAETR	LK
	SQKEDKYEEEIKVLTDKLKEAETRAE	LK
	TIDDLEDELYAQK	LY; EL
	VAKLEKTIDDLEDELY	LY; EL
	VEEELDRAQERLATALTKLEEAEKAADESERGMK	EL
	VEEELDRAQERLATALTKLEEAEKAADESERG	EL
	VRNDEELNKLLGGVTIAQGGVLPN	EL
	VTIMPKDIQLAR	KD

A: alanine; C: cysteine; D: aspartic acid; E: glutamic acid; F: phenylalanine; G: glycine; H: histidine; I: isoleucine; K: lysine; L: leucine; M: methionine; N: asparagine; P: proline; Q: glutamine; R: arginine; S: serine; T: threonine; V: valine; W: tryptophan; Y: tyrosine.

## Data Availability

The datasets generated for this study are available on request to the corresponding author.

## Supplementary Materials

The following supporting information can be downloaded at: https://www.mdpi.com/article/10.3390/foods12142717/s1, Figure S1: Response surface plots for protein recovery (a), TEAC (b) and ORAC (c) values obtained for head extracts, and desirability degree (d). Desirability is based on the common response of the various responses analyzed. The less significant parameter was fixed at its optimal condition.; Figure S2: Response surface plots for protein recovery (a), TEAC (b) and ORAC (c) values obtained for skin extracts, and desirability degree (d). Desirability is based on the common response of the various responses analyzed. The less significant parameter was fixed at its optimal condition. Figure S3: Response surface plots for protein recovery (a), TEAC (b) and ORAC (c) values obtained for viscera extracts, and desirability degree (d). Desirability is based on the common response of the various responses analyzed. The less significant parameter was fixed at its optimal condition. Figure S4: Response surface plots for protein recovery (a), TEAC (b) and ORAC (c) values obtained for backbone extracts, and desirability degree (d). Desirability is based on the common response of the various responses analyzed. The less significant parameter was fixed at its optimal condition. Table S1: Specific energy (kJ/kg), field strength (kV/cm), and time of extraction (h) conditions for each experiment of the response-surface optimization design. Table S2: Protein recovery, TEAC, and ORAC results for sea bass head extract obtained for each experiment of response-surface optimization. Table S3: Protein recovery, TEAC, and ORAC results for sea bass skin extract obtained for each experiment of response-surface optimization. Table S4: Protein recovery, TEAC, and ORAC results for sea bass viscera extract obtained for each experiment of response-surface optimization. Table S5: Protein recovery, TEAC, and ORAC results for sea bass backbone extract obtained for each experiment of response-surface optimization.
