# Supplementary material for: Sea Bass Side Streams Extracts Obtained by Pulsed Electric Fields: Nutritional Characterization and Effect on SH-SY5Y Cells
Source: Foods. 2023 Jul 16;12(14):2717. doi: 10.3390/foods12142717 (PMC10378982; doi:10.3390/foods12142717)
Supplement: Supplementary file 1 [file foods-12-02717-s001.zip › foods-2452697-supplementary.pdf]

foods

MDPI

Article

Sea Bass Side Stream Extracts Obtained by Pulsed Electric Fields: Nutritional Characterization and Effect in SH-SY5Y Cells

Francisco J. Martí-Quijal[1,2], Juan Manuel Castagnini[1,*], María José Ruiz[2], Francisco J. Barba[1,*]

[1] Research group in Innovative Technologies for Sustainable Food (ALISOST), Nutrition, Food Science and Toxicology Department, Faculty of Pharmacy, Universitat de València, Avda. Vicent Andrés Estellés, s/n, 46100, Burjassot, València, Spain; francisco.j.marti@uv.es (F.J.M-Q.); juan.castagnini@uv.es (J.M.C.); francisco.barba@uv.es (F.J.B.)

[2] Research group in Alternative methods for determining toxics effects and risk assessment of contaminants and mixtures (RiskTox), Preventive Medicine and Public Health, Food Science, Toxicology and Forensic Medicine Department, Faculty of Pharmacy, Universitat de València, Avda. Vicent Andrés Estellés, s/n, 46100, Burjassot, València, Spain; francisco.j.marti@uv.es (F.J.M-Q.); m.jose.ruiz@uv.es (M.J.R.)

* Correspondence: juan.castagnini@uv.es (J.M.C.); francisco.barba@uv.es (F.J.B.)

Abstract: Fish side streams are an environmental and economic problem. In this work, pulsed electric fields (PEF) extraction was optimized and used as a new way for their valorization. Sea bass head, skin, viscera and backbone were used for the study. PEF technology improved the extraction of proteins and antioxidant compounds from head and skin, while it was not successful for viscera. SDS-PAGE showed that the protein molecular weight distribution was affected by the extraction process, revealing differences between the control and PEF extraction conditions. In addition, the extraction of macro-minerals and micro-minerals were also evaluated. The effect of PEF on this parameter differed according to the matrix and the mineral studied. Heavy metals were also taken into account, studying the presence of As, Cd, Hg and Pb in the extracts. PEF pre-treatment reduced the presence of As in skin, viscera and backbone, ranging from 18.25 to 28.48%, according to the matrix evaluated. The potential antioxidant bioactive peptides were also analysed, revealing that the treatment of the sample directly affects to the variety of them. Finally, the effect of the extracts on cell viability was assessed using SH-SY5Y cells, observing an increase in cell viability for the extracts obtained from head.

Keywords: pulsed electric fields; fish side streams; antioxidants; minerals; heavy metals; bioactive peptides, cell viability, SH-SY5Y

foods

Table S1. Specific energy (kJ/kg), field strength (kV/cm) and time of extraction (h) conditions for each experiment of the response-surface optimization design.

Experiment	Specific energy (kJ/Kg)	Field strength (kV/cm)	Time of extraction (h)
**1**	300	2	12
**2**	300	1	24
**3**	50	3	24
**4**	50	2	12
**5**	50	1	0
**6**	175	3	12
**7**	175	2	0
**8**	300	1	0
**9**	300	3	0
**10**	50	3	0
**11**	300	3	24
**12**	50	1	24
**13**	175	1	12
**14**	175	2	24
**15**	175	2	12
**16**	175	2	12

Table S2. Protein recovery, TEAC and ORAC results for sea bass head extract obtained for each experiment of response-surface optimization.

Experiment	Specific energy (kJ/Kg)	Field strenght (kV/cm)	Time of extraction (h)	Δ Conductivity (µS/cm·kg)	Δ Temperature (°C/kg)	% Protein Recovery (g/100 g proteins in sample)	Antioxidant Capacity	
							TEAC (µmol TE/L)	ORAC (µmol TE/L)
1	300	2	12	68.1	1.6	45.73	339.40	341.21
2	300	1	24	98.6	18.0	78.28	432.16	514.85
3	50	3	24	69.6	13.5	55.51	300.37	415.51
4	50	2	12	28.5	0.4	46.98	339.40	265.58
5	50	1	0	22.4	5.9	27.00	91.64	32.00
6	175	3	12	132.1	41.0	64.23	347.69	418.80
7	175	2	0	183.0	1.4	42.43	73.09	36.51
8	300	1	0	96.8	17.8	35.63	162.53	98.09
9	300	3	0	172.0	60.9	35.67	124.63	147.47
10	50	3	0	78.2	15.0	32.92	52.50	18.63
11	300	3	24	244.7	55.0	89.63	343.65	432.61
12	50	1	24	22.9	5.6	46.22	405.26	302.77
13	175	1	12	43.8	12.9	67.80	593.59	625.19
14	175	2	24	51.3	1.9	61.56	439.79	576.80
15	175	2	12	70.3	0.3	58.04	421.09	320.57
16	175	2	12	60.9	1.2	51.90	401.85	438.31

foods

MDPI

Table S3. Protein recovery, TEAC and ORAC results for sea bass skin extract obtained for each experiment of response-surface optimization.

Experiment	Specific energy (kJ/Kg)	Field strenght (kV/cm)	Time of extraction (h)	Δ Conductivity (µS/cm·kg)	Δ Temperature (°C/kg)	% Protein Recovery (g/100 g proteins in sample)	Antioxidant Capacity	
							TEAC (µmol TE/L)	ORAC (µmol TE/L)
1	300	2	12	300.5	5.2	12.88	505.92	721.93
2	300	1	24	208.6	20.3	29.93	645.70	613.07
3	50	3	24	217.4	28.8	13.87	543.90	442.68
4	50	2	12	176.8	1.7	13.55	490.88	881.63
5	50	1	0	91.6	6.0	6.72	119.85	158.65
6	175	3	12	330.1	61.9	20.58	551.59	604.00
7	175	2	0	201.4	5.0	5.54	120.02	146.01
8	300	1	0	159.8	21.5	10.56	166.17	441.91
9	300	3	0	462.8	91.5	8.88	181.52	435.32
10	50	3	0	129.0	22.9	5.09	52.10	52.60
11	300	3	24	239.1	92.9	39.58	749.28	1662.31
12	50	1	24	120.8	6.7	12.61	265.16	285.03
13	175	1	12	85.9	15.0	16.72	435.50	917.60
14	175	2	24	155.8	4.8	13.36	486.52	429.35
15	175	2	12	290.5	3.9	10.39	445.07	566.29
16	175	2	12	131.1	3.7	11.46	440.67	330.07

Table S4. Protein recovery, TEAC and ORAC results for sea bass viscera extract obtained for each experiment of response-surface optimization.

Experiment	Specific energy (kJ/Kg)	Field strenght (kV/cm)	Time of extraction (h)	Δ Conductivity (μS/cm·kg)	Δ Temperature (˚C/kg)	% Protein Recovery (g/100 g proteins in sample)	Antioxidant Capacity	
							TEAC (μmol TE/L)	ORAC (μmol TE/L)
1	300	2	12	203.2	2.7	51.85	894.44	2262.10
2	300	1	24	161.7	20.4	46.23	648.91	1358.19
3	50	3	24	90.9	9.7	53.93	952.99	2316.29
4	50	2	12	36.4	0.4	97.31	1041.30	2927.15
5	50	1	0	25.6	6.3	20.14	173.44	161.82
6	175	3	12	266.8	29.0	107.25	1129.73	5680.53
7	175	2	0	278.5	0.7	23.83	341.66	199.51
8	300	1	0	137.8	20.2	32.81	568.90	707.21
9	300	3	0	290.3	47.5	23.74	452.11	424.72
10	50	3	0	90.9	10.3	18.40	139.58	78.45
11	300	3	24	426.2	49.7	33.27	1046.67	794.38
12	50	1	24	45.7	5.7	52.85	701.03	2218.30
13	175	1	12	74.0	14.0	49.77	983.51	2482.46
14	175	2	24	49.3	1.2	40.20	1020.99	1926.99
15	175	2	12	30.1	0.8	72.17	921.18	3064.80
16	175	2	12	49.8	2.8	83.44	922.91	2427.66

Table S5. Protein recovery, TEAC and ORAC results for sea bass backbone extract obtained for each experiment of response-surface optimization.

Experiment	Specific energy (kJ/Kg)	Field strenght (kV/cm)	Time of extraction (h)	Δ Conductivity (μS/cm·kg)	Δ Temperature (˚C/kg)	% Protein Recovery (g/100 g proteins in sample)	Antioxidant Capacity	
							TEAC (μmol TE/L)	ORAC (μmol TE/L)
1	300	2	12	262.0	4.4	25.43	1.14	749.68
2	300	1	24	234.9	19.2	28.03	23.00	544.48
3	50	3	24	179.4	20.9	25.41	9.76	469.19
4	50	2	12	53.1	3.7	29.72	8.65	669.95
5	50	1	0	65.6	6.7	12.90	13.28	111.31
6	175	3	12	237.2	47.3	28.75	0.07	443.35
7	175	2	0	117.2	4.2	10.89	10.09	46.59
8	300	1	0	165.0	22.7	15.76	11.39	167.48
9	300	3	0	289.9	65.0	13.61	8.49	154.37
10	50	3	0	177.8	17.6	12.73	9.92	92.66
11	300	3	24	314.0	64.8	24.91	0	259.42
12	50	1	24	155.0	6.6	23.37	18.60	382.67
13	175	1	12	62.4	16.9	27.92	1.22	746.61
14	175	2	24	232.5	2.5	25.55	14.61	494.11
15	175	2	12	181.4	3.0	29.17	21.35	633.47
16	175	2	12	137.5	1.3	21.77	11.93	391.30

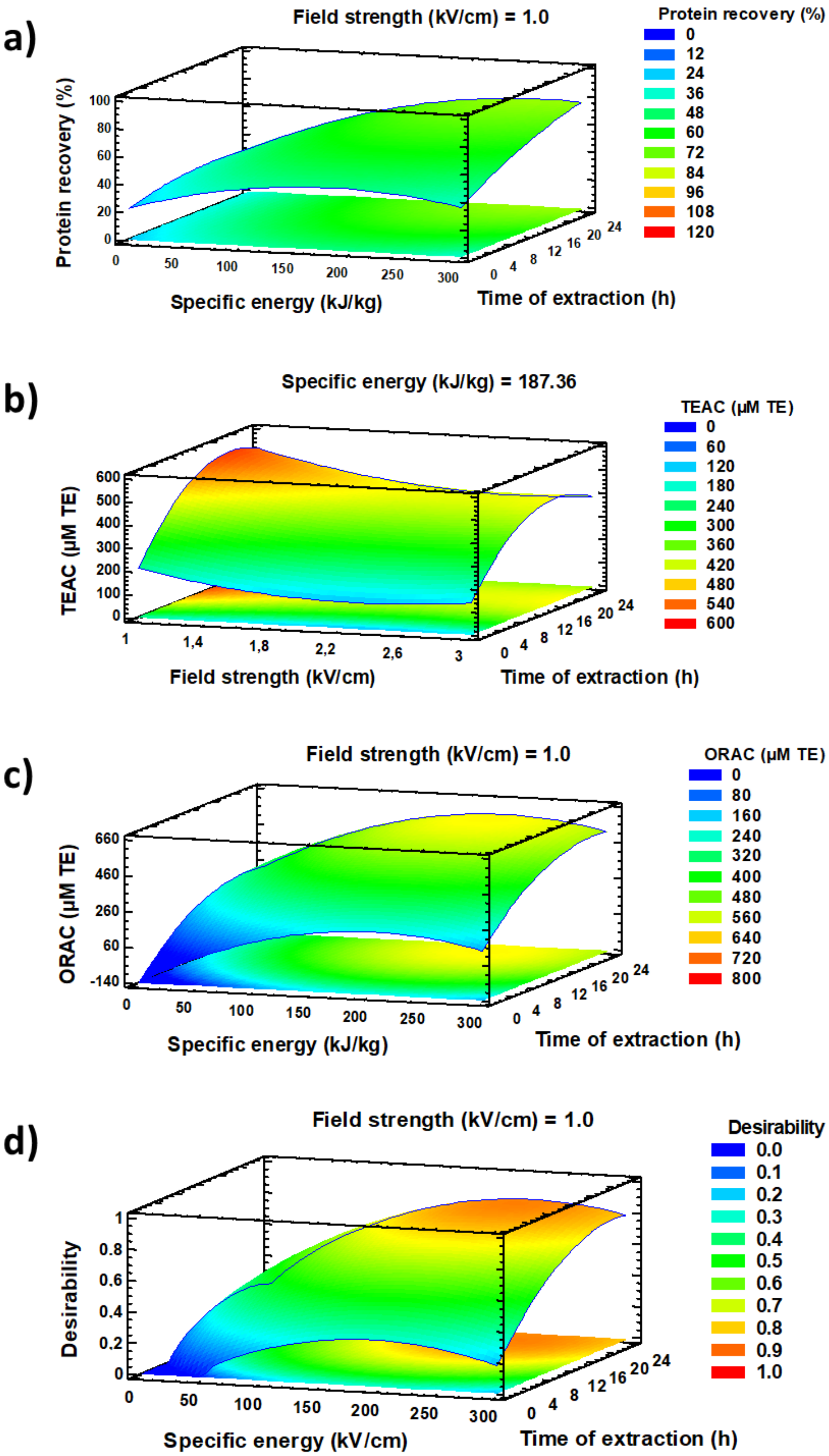

Figure S1. Response surface plots for protein recovery (a), TEAC (b) and ORAC (c) values obtained for head extracts and desirability degree (d). Desirability is based on the common response of the various responses analyzed. The less significant parameter was fixed at its optimal condition

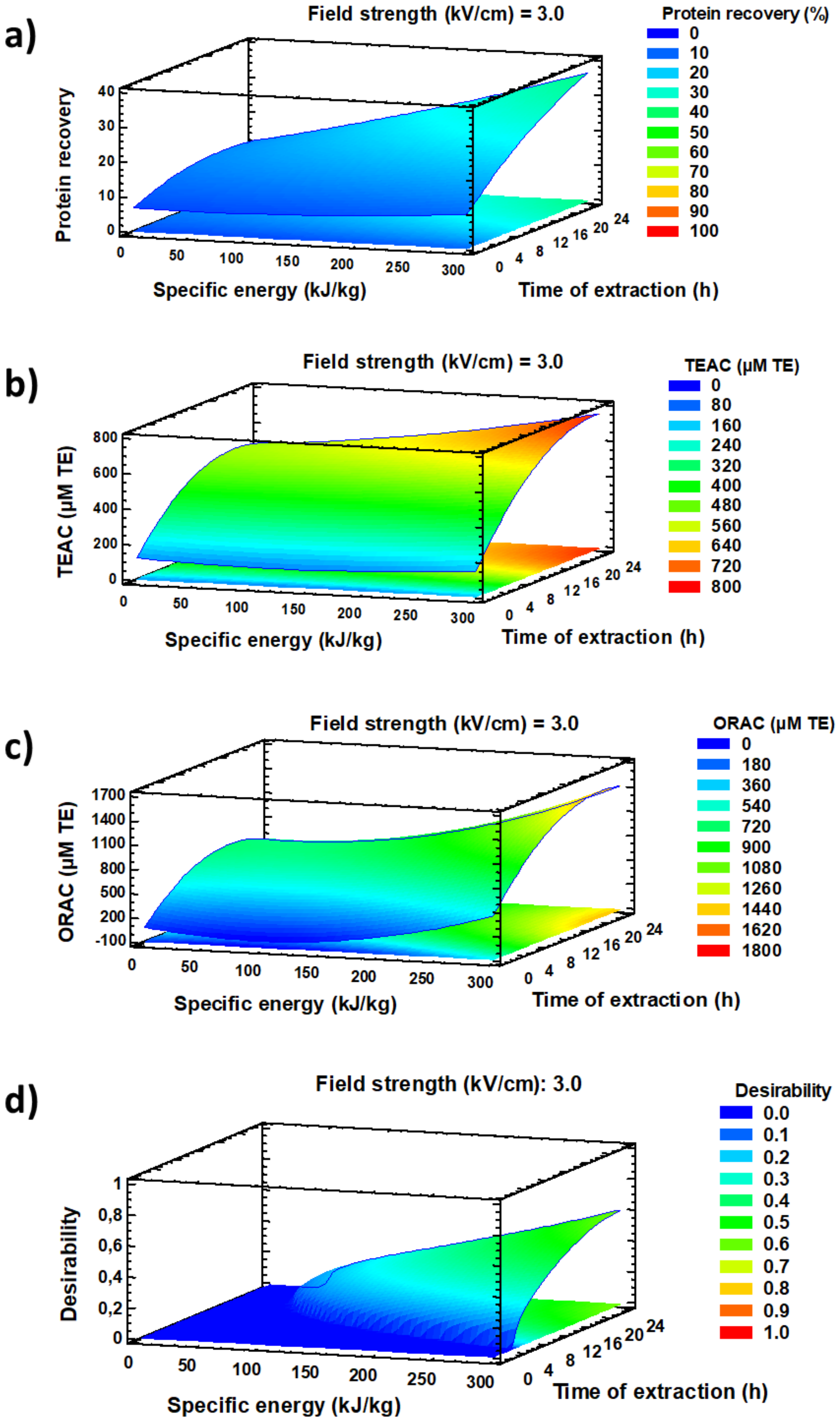

Figure S2. Response surface plots for protein recovery (a), TEAC (b) and ORAC (c) values obtained for skin extracts and desirability degree (d). Desirability is based on the common response of the various responses analyzed. The less significant parameter was fixed at its optimal condition

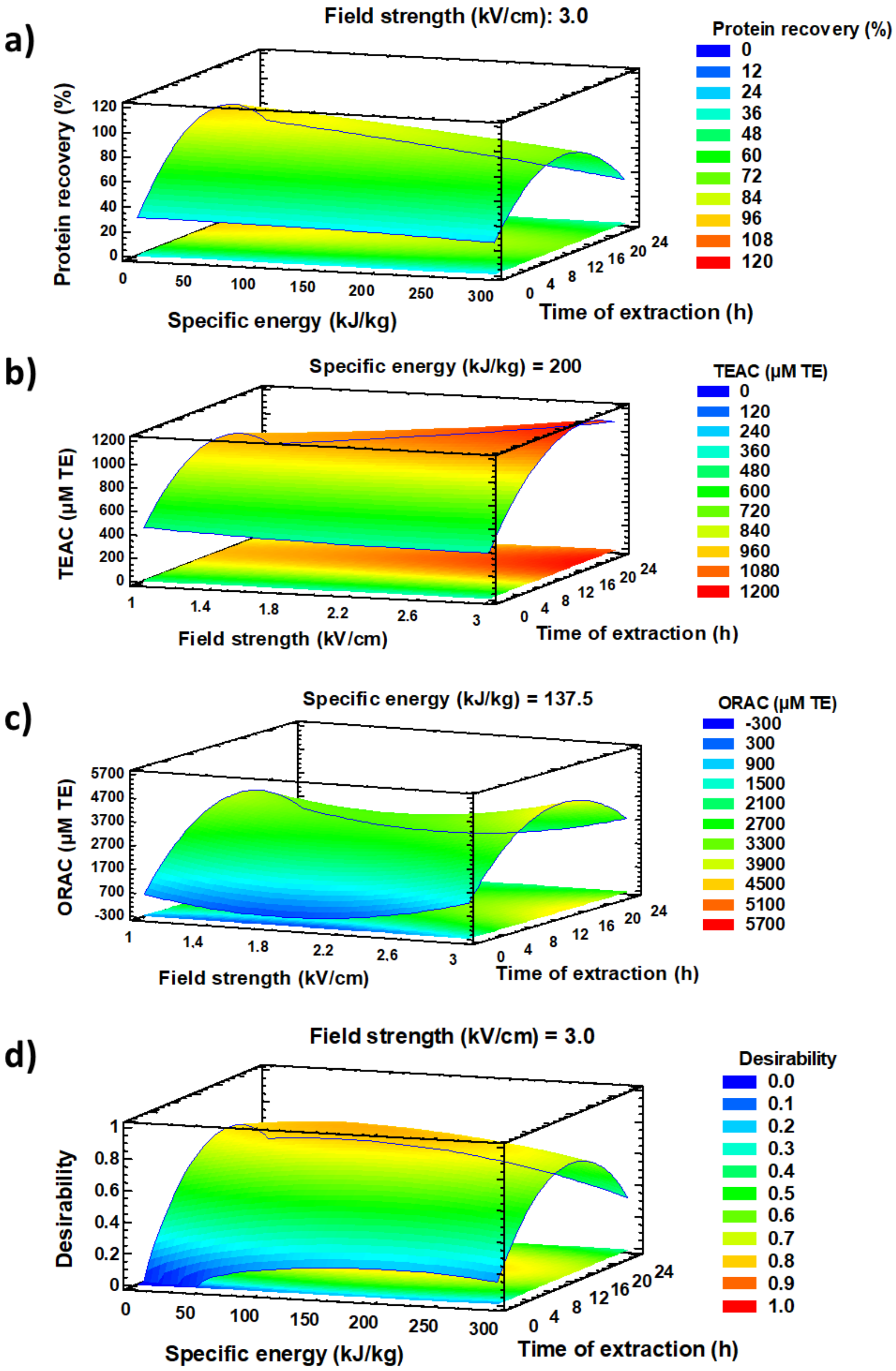

Figure S3. Response surface plots for protein recovery (a), TEAC (b) and ORAC (c) values obtained for viscera extracts and desirability degree (d). Desirability is based on the common response of the various responses analyzed. The less significant parameter was fixed at its optimal condition

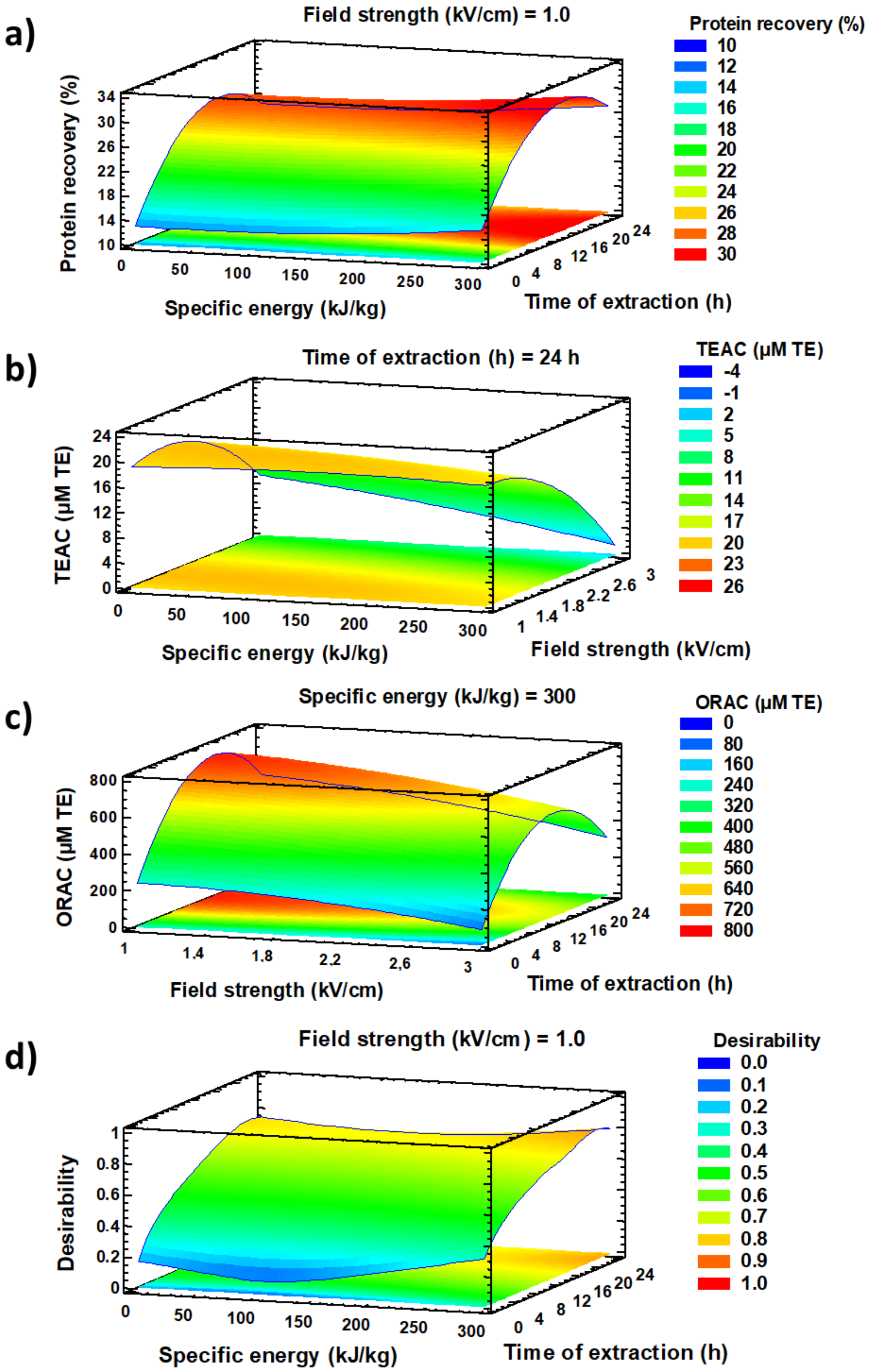

Figure S4. Response surface plots for protein recovery (a), TEAC (b) and ORAC (c) values obtained for backbone extracts and desirability degree (d). Desirability is based on the common response of the various responses analyzed. The less significant parameter was fixed at its optimal condition.
